# The Imperative of Trauma-Informed Care: A Comprehensive Review and Strategies for Implementation in Health Services

**DOI:** 10.1192/j.eurpsy.2024.1699

**Published:** 2024-08-27

**Authors:** C. Cunha, M. Gomes

**Affiliations:** ^1^Hospital de Magalhaes Lemos, Porto; ^2^Hospital de Braga, Braga, Portugal

## Abstract

**Introduction:**

Psychological trauma is a significant public health concern with long-lasting effects on physical and mental well-being. Trauma-informed care is an approach to providing support and services that acknowledges and integrates an understanding of the pervasive impact of trauma on individuals. This review delves into the critical imperative of trauma-informed care within the realm of health services. Recognizing the pervasive impact of trauma on individuals’ physical and mental well-being, this REVIEW aims to explore existing literature, identify key objectives, and propose effective methods for implementing trauma-informed strategies in health services.

**Objectives:**

To Review Existing Literature on Trauma: Conduct an review of the literature to comprehend the varied dimensions and consequences of trauma on individuals’ health; To Identify Key Principles of Trauma-Informed Care: Explore established principles of trauma-informed care, highlighting their relevance and applicability within health service settings; To Propose Implementation Strategies: Develop practical strategies for integrating trauma-informed care into health services, ensuring a comprehensive and sensitive approach to patient care.

**Methods:**

A review of published articles, books, and reports related to trauma and trauma-informed care to establish a foundational understanding.

**Results:**

Psychological trauma can have profound and multifaceted impacts on individuals, affecting their mental, emotional, and even physical well-being. The consequences of psychological trauma can vary widely based on the nature, severity, and duration of the traumatic experience, as well as individual factors such as resilience and support systems. Trauma-informed care aims to create an environment that is sensitive to the needs of those who have experienced trauma, and it is based on six key principles: safety, trustworthiness, peer support, collaboration, empowerment, and cultural competence. Healthcare providers need to understand trauma beyond the personal and acknowledge the cultural, historical, social, political, and structural trauma that impact individuals and communities across generations. This approach recognizes that there is a risk of retraumatization in social and health services, especially for minority communities.

**Conclusions:**

This review underscores the pressing need for health services to adopt trauma-informed care strategies. By acknowledging the prevalence and impact of trauma on health outcomes, the healthcare sector can transition towards a more patient-centered and empathetic approach.

**Disclosure of Interest:**

None Declared

